# Effectiveness of a self-instructional module on home care management of diabetes mellitus among adults

**DOI:** 10.6026/973206300210578

**Published:** 2025-04-30

**Authors:** Beula S.S, Siva Subramanian N., Mahalakshmi B., Patel Princekumar Satishkumar, Patel Pransi Baldevbhai, Patel Mihir Jigarkumar, Karan Vikrambhai Chaudhary, Roshankumar Dineshbhai Patel, Patel Sakshi Maheshkumar, Patel Meshva Rajeshkumar

**Affiliations:** 1 Department of Community Health Nursing, Nootan College of Nursing, Sankalchand Patel University, Visnagar, Gujarat - 384315; India; 2 Department of Psychiatric Nursing, Nootan College of Nursing, Sankalchand Patel University, Visnagar, Gujarat - 384315; India; 3 Department of Paediatric Nursing, Nootan College of Nursing, Sankalchand Patel University, Visnagar, Gujarat - 384315; India

**Keywords:** Effectiveness, self-instructional module, home care management, diabetes mellitus

## Abstract

The effectiveness of a self-instructional module (SIM) on knowledge regarding home care management of diabetes mellitus among adults in Visnagar is of interest. A pre-experimental one-group pre-test and post-test design was used, with 50 diabetic patients selected through non-probability convenience sampling. A structured questionnaire assessed knowledge before and after the intervention. The results showed a significant improvement in post-test scores, demonstrating the effectiveness of SIM in enhancing diabetes-related knowledge. The mean pre-test score was 12.5 ± 3.2, which increased to 20.8 ± 2.8 post-intervention (p < 0.05). Thus, SIM is an effective tool for diabetes education, especially in low-resource settings and can significantly improve self-care practices and disease management among diabetic patients.

## Background:

Diabetes mellitus (DM) is a chronic metabolic disorder that requires continuous management to prevent complications and improve the quality of life for affected individuals. The prevalence of DM is increasing globally, with developing nations witnessing a significant rise due to lifestyle changes, urbanization and sedentary behavior. Studies have shown that structured home care interventions can enhance diabetes self-management, leading to better glycemic control and reduced risk of complications [1]. Home-based diabetes care programs, such as structured education and self-monitoring techniques, have been found to significantly improve regulation in type 2 diabetes patients. Research highlights that self-monitoring blood glucose (SMBG) as part of home care interventions can enhance patient adherence to dietary modifications, medication use and lifestyle changes, ultimately improving diabetes outcomes [2]. A study conducted on home-based nursing care programs demonstrated significant reductions in fasting blood glucose, hemoglobin A1C and triglyceride levels, reinforcing the importance of home interventions in managing diabetes [3]. Innovative healthcare-supportive home systems have also shown promise in improving diabetes self-care. Studies suggest that digital health integration, such as automated monitoring and remote consultations, can empower patients to maintain optimal glucose levels and reduce the need for frequent hospital visits [4]. Another study highlights that home care counselling plays a crucial role in diabetes management by offering psychological and emotional support alongside medical interventions, leading to improved patient engagement and adherence to treatment plans [5]. Additionally, nursing care interventions at home have been shown to positively impact metabolic control in diabetes patients. A randomized controlled trial reported significant improvements in glycemic control, lipid profiles and overall health outcomes in individuals who received structured nursing care at home compared to standard outpatient treatment [6]. Furthermore, research has emphasized the importance of family involvement in home diabetes care, as family members play a crucial role in supporting patients with medication adherence, diet and lifestyle modifications [7]. Given the increasing burden of diabetes, self-instructional modules (SIMs) have emerged as a practical educational tool to enhance patient knowledge regarding home care management. Studies have found that structured educational interventions significantly improve self-care behaviors and glycemic control, particularly in patients receiving home-based diabetes education [8]. Therefore, it is of interest to assess the effectiveness of self-instructional modules in improving diabetes home care knowledge and management among adults in selected urban areas of Visnagar.

## Methodology:

## Research design and setting:

This study employed a pre-experimental one-group pre-test and post-test design to assess the effectiveness of a self-instructional module (SIM) on home care management of diabetes mellitus. The study was conducted in selected urban areas of Visnagar, India, where a significant number of adults diagnosed with diabetes mellitus reside.

## Population and sample:

The target population included adults aged 30-50 years diagnosed with diabetes mellitus residing in Visnagar. A total of 50 participants were selected using a non-probability convenience sampling technique.

## Inclusion and Exclusion criteria:

Participants were included if they were 30-50 years old, diagnosed with diabetes, understood Gujarati and were willing to participate. Those with severe diabetes complications, unwilling to participate, or unable to read Gujarati were excluded.

## Development of the research tool:

The tool consisted of three sections: Section A (demographic variables), Section B (structured knowledge questionnaire on diabetes home care) and Section C (Self-Instructional Module covering diet, medication, glucose monitoring and lifestyle modifications). Content validity was established by six experts, including healthcare professionals and reliability was tested using the split-half method, yielding a Cronbach's alpha score of >0.80, indicating high reliability.

## Data collection procedure:

A pre-test was conducted using a structured questionnaire, followed by the distribution of the Self-Instructional Module (SIM) to participants. After one week, a post-test was administered using the same questionnaire to assess knowledge improvement. Approval was obtained from the Ethical Committee of Nootan College of Nursing, Visnagar and informed consent was taken from all participants. Confidentiality was maintained and participants could withdraw at any stage.

## Data analysis:

Descriptive statistics such as frequency, percentage, mean and standard deviation were used to analyze demographic data and knowledge scores. A paired t-test was used to compare pre-test and post-test scores and a chi-square test determined the association between knowledge improvement and demographic variables.

## Results:

Table 1 shows the demographic distribution of diabetic patients, indicating that the majority are aged 41-50 years, male and have secondary education. Table 2 shows that the mean post-test knowledge score is significantly higher than the pre-test score, demonstrating the effectiveness of the Self-Instructional Module. Table 3 shows a significant association between knowledge levels and factors like age, education level and occupation, while gender and duration of diabetes show no significant association. Figure 1 - (see PDF) shows a significant improvement in knowledge levels, with the number of participants in the "good" category increasing from 10% to 60% after the intervention.

## Discussion:

Evaluate the effectiveness of a self-instructional module (SIM) on improving knowledge regarding home care management of diabetes mellitus among adults in Visnagar. The results indicated a significant improvement in post-test knowledge scores, demonstrating the effectiveness of SIM as an educational intervention. Several studies support the effectiveness of structured educational interventions in diabetes management. A systematic review and meta-analysis found that pharmacist-led educational programs significantly improved self-care behaviors, including blood glucose monitoring, dietary adherence and foot care, leading to a reduction in HbA1c levels [9]. Similarly, a study assessing nurse-led educational interventions reported improved glycemic control and self-care behaviors, suggesting that structured education significantly enhances diabetes self-management [10].

The effectiveness of educational interventions is also evident in elderly populations. A quasi-experimental study found that group education programs led to significant improvements in dietary habits, foot care and overall diabetes self-care behaviors [11]. Similarly, a randomized controlled trial (RCT) on low-education diabetic patients demonstrated that structured communication-based educational interventions significantly reduced HbA1c levels [12]. Our findings also align with theory-based educational interventions in diabetes management. A study using the Health Belief Model (HBM) demonstrated that educational interventions significantly improved self-care behaviors, medication adherence and glycemic control [13]. Similarly, interventions based on the Theory of Planned Behavior (TPB) were effective in improving self-care behaviors, reducing HbA1c and increasing diabetes-related knowledge [14]. The mode of delivery of educational interventions also plays a critical role. Studies have shown that mobile-based interventions (SMS messages) can significantly enhance self-care behaviors in diabetic patients [15]. Another study comparing animation and role-playing educational interventions found that animation-based self-care education led to greater improvements in quality of life [16]. These findings suggest that engaging, interactive educational tools may enhance learning and behavior change. Our study demonstrated a significant improvement in post-test knowledge scores after implementing a Self-Instructional Module (SIM), aligning with multiple previous studies.

Athawale & Thombare (2023) found that diabetic patients had poor pre-test knowledge, with no participants scoring in the good category, but post-test scores improved by 28.37%, proving SIM's effectiveness in diabetes education [17]. Kharel (2023) found that 60% of diabetic patients had inadequate knowledge before SIM, but 68.3% attained adequate knowledge post-intervention, reinforcing SIM's effectiveness in self-care management [18]. Pawar (2023) reported a knowledge improvement from 58.51% to 84.13% among Type II diabetes patients post-SIM, with a significant statistical difference (t=3.64, p≤0.001), highlighting its role in self-care education [19]. Systematic reviews by Ernawati *et al.* (2021) and [21] Sangeeta *et al.* (2019) confirmed that structured diabetes self-management education (DSME) significantly enhances self-care, dietary adherence and glycemic control, supporting our findings on the effectiveness of structured education for diabetes patients [20, 21]. Our study further supports the role of structured self-care education in improving diabetes management, as observed in meta-analysis comparing various educational models also found that self-efficacy-based structured education significantly improved diabetes-related knowledge and self-care [22]. In conclusion, our findings align with a growing body of evidence supporting the effectiveness of structured educational interventions in improving diabetes self-care, self-efficacy and glycemic control. Given the success of SIM in our study and similar results in previous research, future interventions should incorporate theory-based, interactive and technology-supported educational models to enhance diabetes self-management and long-term health outcomes.

## Figures and Tables

**Table 1:** Frequency and percentage distribution of socio-demographic variables of diabetic patients (N=50)

**Variable**	**Category**	**Frequency (n)**	**Percentage (%)**
Age (Years)	30-40	20	40%
	41-50	30	60%
Gender	Male	28	56%
	Female	22	44%
Education Level	No Formal Education	5	10%
	Primary	10	20%
	Secondary	20	40%
	Higher Education	15	30%
Occupation	Employed	18	36%
	Unemployed	32	64%
Duration of Diabetes (Years)	<5	15	30%
	10-May	25	50%
	>10	10	20%

**Table 2:** Mean, Standard deviation and t-test of pre-test and post-test knowledge scores of diabetic patients (N=50)

**Test**	**Mean (M)**	**Standard Deviation (SD)**	**t-value**	**p-value**
Pre-Test	12.5	3.2	10.98	0.0001*
Post-Test	20.8	2.8		
(P < 0.05 indicates statistical significance)

**Table 3:** Association between knowledge levels and selected socio-demographic variables of diabetic patients (N=50)

**Variable**	**Chi-Square (χ^2^)**	**-value**	**Significance**
Age	4.56	0.02*	Significant
Gender	1.32	0.25	Not Significant
Education Level	6.89	0.001*	Significant
Occupation	3.45	0.04*	Significant
Duration of Diabetes	2.98	0.09	Not Significant
(P < 0.05 indicates statistical significance)
